# Reduced dosage of *β-catenin* provides significant rescue of cardiac outflow tract anomalies in a *Tbx1* conditional null mouse model of 22q11.2 deletion syndrome

**DOI:** 10.1371/journal.pgen.1006687

**Published:** 2017-03-27

**Authors:** Silvia E. Racedo, Erica Hasten, Mingyan Lin, Gnanapackiam Sheela Devakanmalai, Tingwei Guo, Ertugrul M. Ozbudak, Chen-Leng Cai, Deyou Zheng, Bernice E. Morrow

**Affiliations:** 1 Department of Genetics, Albert Einstein College of Medicine, Bronx, NY, United States of America; 2 Department of Developmental and Regenerative Biology, Icahn School of Medicine at Mount Sinai, New York, NY, United States of America; 3 Department of Neurology, Albert Einstein College of Medicine, Bronx, NY, United States of America; 4 Department of Neuroscience, Albert Einstein College of Medicine, Bronx, NY, United States of America; Stanford University School of Medicine, UNITED STATES

## Abstract

The 22q11.2 deletion syndrome (22q11.2DS; velo-cardio-facial syndrome; DiGeorge syndrome) is a congenital anomaly disorder in which haploinsufficiency of *TBX1*, encoding a T-box transcription factor, is the major candidate for cardiac outflow tract (OFT) malformations. Inactivation of *Tbx1* in the anterior heart field (AHF) mesoderm in the mouse results in premature expression of pro-differentiation genes and a persistent truncus arteriosus (PTA) in which septation does not form between the aorta and pulmonary trunk. Canonical *Wnt/β-catenin* has major roles in cardiac OFT development that may act upstream of *Tbx1*. Consistent with an antagonistic relationship, we found the opposite gene expression changes occurred in the AHF in *β-catenin* loss of function embryos compared to *Tbx1* loss of function embryos, providing an opportunity to test for genetic rescue. When both alleles of *Tbx1* and one allele of *β-catenin* were inactivated in the *Mef2c-AHF-Cre* domain, 61% of them (n = 34) showed partial or complete rescue of the PTA defect. Upregulated genes that were oppositely changed in expression in individual mutant embryos were normalized in significantly rescued embryos. Further, *β-catenin* was increased in expression when *Tbx1* was inactivated, suggesting that there may be a negative feedback loop between canonical Wnt and *Tbx1* in the AHF to allow the formation of the OFT. We suggest that alteration of this balance may contribute to variable expressivity in 22q11.2DS.

## Author summary

To understand the genetic relationship between Tbx1 and canonical *Wnt*/*β-catenin*, we performed gene expression profiling and genetic rescue experiments. We found that *Tbx1* and *β-catenin* may provide a negative feedback loop to restrict premature differentiation in the anterior heart field. This is relevant to understanding the basis of variable expressivity of 22q11.2DS, caused by haploinsufficiency of *TBX1*.

## Introduction

The 22q11.2 deletion syndrome (22q11.2DS), also known as velo-cardio-facial syndrome (MIM# 192430) or DiGeorge syndrome (MIM# 188400) is a congenital malformation disorder that is caused by a hemizygous 1.5–3 million base pair (Mb) deletion of chromosome 22q11.2. It occurs with a frequency of 1:1,000 fetuses [1] and 1:4,000 live births [2]. Approximately 60–70% of affected 22q11.2DS individuals have congenital heart disease (CHD) due to malformations of the aortic arch and/or cardiac outflow tract [3]. There are over 46 known coding genes in the 3 Mb region, including *TBX1* (T-box 1; MIM# 602054), encoding a T-box containing transcription factor [4]. *TBX1* has been considered the strongest candidate gene for CHD, based upon studies of mouse models [5–7] and discovery of mutations in some non-deleted patients [8, 9]. The basis of variable phenotypic expression is under intense investigation. Understanding responsible genetic factors upstream and downstream of TBX1 is necessary to test for relevancy as modifiers in human 22q11.2DS patients. We are taking mouse genetic approaches to identify genes and networks that may act as modifiers.

*Tbx1* heterozygous mice have mild aortic arch anomalies or ventricular septal defects, at reduced penetrance, while all homozygous null mutant mice die at birth and have a persistent truncus arteriosus (PTA), which is the most serious heart defect that occurs in 22q11.2DS patients [5–7]. In mammals, *Tbx1* is expressed strongly in the embryonic pharyngeal apparatus, but not the heart tube itself suggesting that its critical functions are in this tissue [4].

In the early vertebrate embryo, the heart forms as a bilateral cardiac crescent of mesodermal cells, termed the first heart field that fuses to form the primitive heart tube [10, 11]. Additional mesodermal cells derived from the pharyngeal apparatus, referred to as the second heart field (SHF) migrates and helps to expand the heart tube in both directions [12] [13] [13–16]. These cells remain in a progenitor state, allowing them to migrate and build the length of the heart tube, where they differentiate into smooth and cardiac muscle and endothelial cells [17, 18]. The SHF itself, can be further subdivided to the anterior heart field (AHF or anterior SHF) forming the cardiac OFT and right ventricle as well as the posterior SHF forming the inflow tract, respectively, based upon gene expression and cell lineage studies [19–21]. Of interest, *Tbx1* is strongly expressed in the pharyngeal mesoderm, including the AHF, but it is not noticeably expressed in the posterior SHF or heart tube [22–24]. One of the key functions of AHF cells is to maintain a progenitor cell state and to prevent premature differentiation. [25] Gene expression profiling of the AHF, within pharyngeal arches two to six, in *Tbx1*^*-/-*^ embryos versus wild type littermates [24] and embryonic stem cell lineage studies [22], suggest that *Tbx1* serves to restrict premature differentiation of the pharyngeal mesoderm, so as to allow the OFT to elongate properly [25]. However, the tissue specificity and key molecular mechanisms are not well defined.

The basis for premature differentiation in the AHF in *Tbx1* mutant embryos is unknown. Major signaling pathways likely have a role in this process. The canonical Wnt signaling pathway is mediated by β-catenin, which has critical functions in most aspects of embryonic development. There are multiphasic functions of Wnt/β-catenin in the pharyngeal mesoderm required for heart development [26]. Several years ago, it was shown that canonical Wnt/β-catenin has a major role in the AHF in forming the cardiac OFT [27]. Further, one study showed that increased or decreased *Wnt/β-catenin* in the pharyngeal mesenchyme (*Dermo*^*Cre*^) resulted in a decrease or increase in *Tbx1* expression, implicating antagonistic functions upstream of *Tbx1* [28]. However, genetic interaction studies were not explored nor were gene expression profiling performed to understand possible molecular connections. Such studies would provide possible modifier genes to investigate in human 22q11.2DS to understand its variable expressivity. In this report we performed genetic rescue experiments between *Tbx1* and *β-catenin* in the AHF, using mouse models.

## Results

### Constitutive *β-catenin* expression in the AHF promotes differentiation

**Fig 1:**
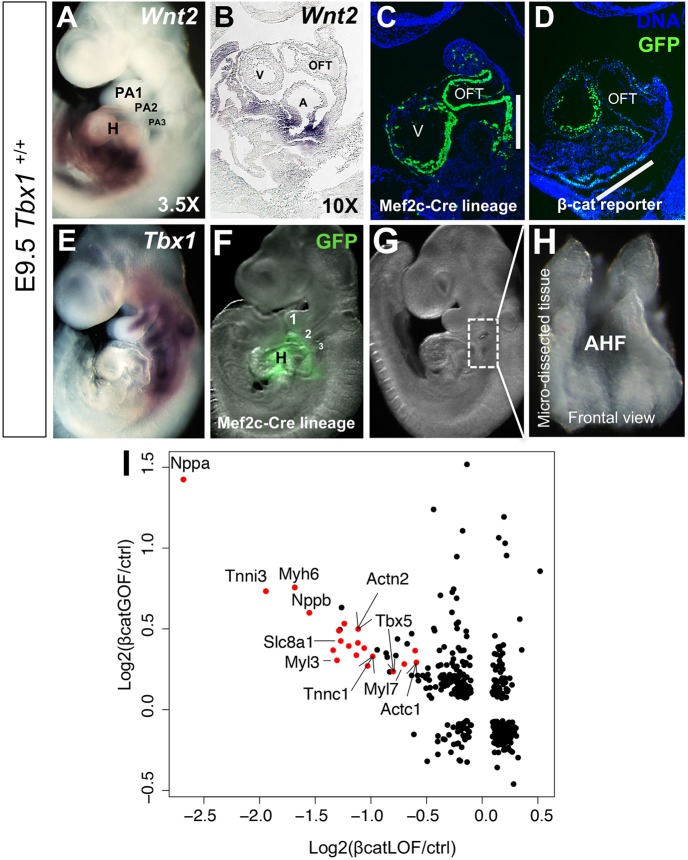
Constitutive *β-catenin* expression in the AHF promotes differentiation. (A) Whole mount *in situ* hybridization (WMISH) of *Wnt2* in a wild type (*Tbx1*^*+/+*^) embryo at embryonic day 9.5 (E9.5) and a sagittal section from the heart region of the embryo is shown in B. (C) *Mef2c-AHF-Cre* lineage in a wild type embryo in a sagittal section. (D) Visualization of β-catenin signaling using a *TCF/Lef*:*H2B-GFP* reporter allele in a sagittal section of a wild type embryo. (E) WMISH of *Tbx1* in a wild type embryo. (F) *Mef2c-AHF-Cre* lineage shown by green fluorescence in a wild type embryo (*Mef2c-AHF-Cre/+;ROSA26-*^*GFP f/+*^) at E9.5. Numbers denote the pharyngeal arches 1, 2 and 3. (G) Whole mount embryo showing the region that has been dissected for the experiments (rectangle in dorsal to the heart). (H) The micro-dissected region containing the *Mef2c-AHF-Cre* lineage is shown from a frontal view. (I) Comparison of global gene expression changes in micro-dissected tissues of *β-catenin GOF* and *β-catenin LOF* embryos at E9.5. Differentially expressed genes (p < 0.05 and FC > 1.5) in at least one of the two comparisons, *β-catenin LOF* vs control (x-axis) or *β-catenin GOF* vs control (y-axis), were plotted. Red dots denote cardiac differentiation genes. Abbreviations: heart (H), pharyngeal arch (PA), outflow tract (OFT), ventricle (V), atrium (A), FC = fold change.

*Wnt/β-catenin* and *Tbx1* are expressed in the opposite domains of the SHF, with *Tbx1* higher in the AHF and *Wnt/β-catenin* higher in the posterior SHF, as denoted by *Wnt2* and *Mef2c-AHF-Cre* [18] lineage compared to canonical Wnt signaling (Fig 1A–1E). We were interested in further exploring the function of *β-*catenin when completely diminished (*Mef2c-AHF-Cre/+;β-catenin*^*flox/flox*^, referred to as *β-cat LOF* [29]) or constitutively active (*Mef2c-AHF-Cre/+;β-catenin*^*Ex3/+*^ [30], referred to as *β-cat GOF*) in the AHF. To identify downstream genes affected by these changes, gene expression profiling was performed on the distal pharyngeal apparatus containing the AHF micro-dissected from *β-cat LOF* and *β-cat GOF* embryos at E9.5 (Fig 1F–1H). Note that the dissection of the AHF did not include the heart tube. In order to highlight the genes with the greatest fold change, we created a dot plot of log2 fold changes (Fig 1I). Loss of both *β-catenin* alleles in the *Mef2c-AHF-Cre* domain resulted in strongly reduced expression of muscle structural genes in the AHF, while constitutive activation of *β-catenin* in this domain, had the opposite effect and caused a strong increase in expression of the same genes in the AHF (Fig 1I). This increase was strikingly similar to that in the AHF of global *Tbx1* null mutant embryos that were previously reported [22, 24, 31]. We then examined cardiac phenotypes upon inactivation of *Tbx1* in the *Mef2c-AHF-Cre* lineage to determine whether *Tbx1* had a specific role in the AHF.

### Persistent truncus arteriosus in *Tbx1 LOF* embryos

**Fig 2:**
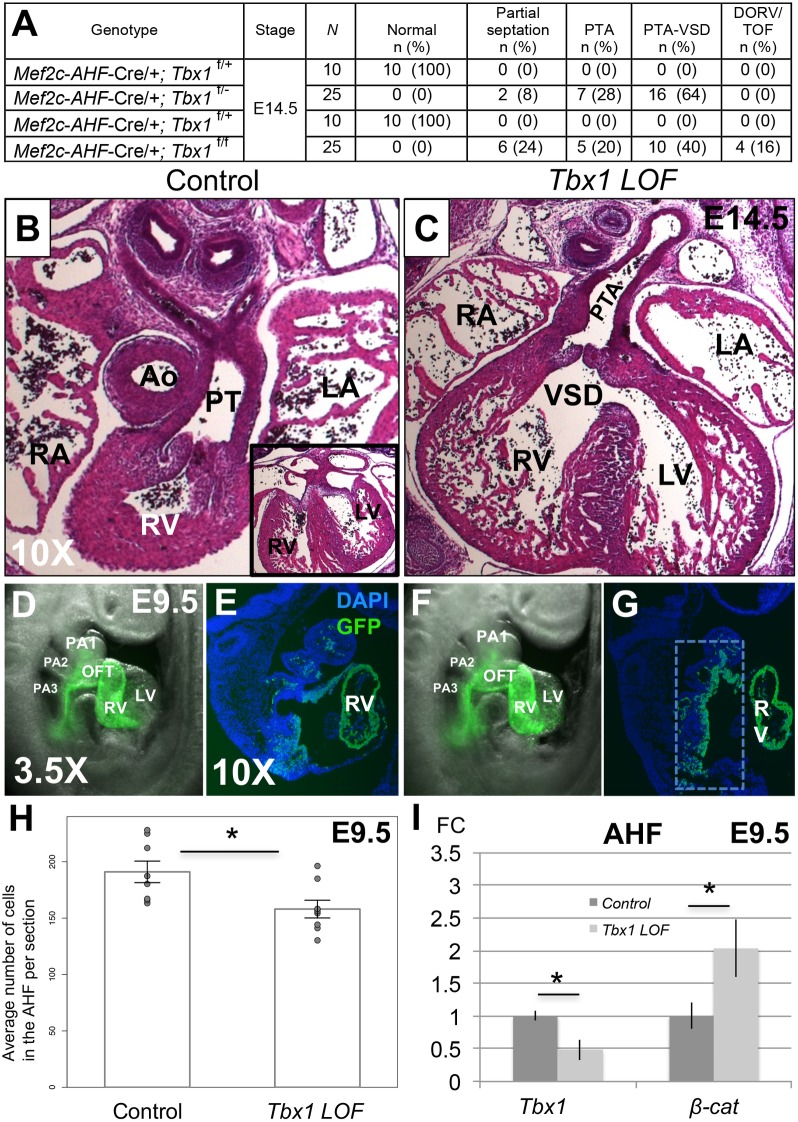
Congenital heart defects in *Tbx1 LOF* embryos. (A) Heart phenotype analysis of *Tbx1 LOF* embryos at E14.5 generated from two different crosses. PTA-VSD refers to a PTA associated with a ventricular septal defect (VSD) while PTA refers to hearts that did not show a VSD. Partial septation in *Tbx1 LOF* embryos means a PTA with presence of a short septum at some level of the OFT and complete septation between the ventricles. N = total number of hearts observed per group. Significance between *Tbx1 LOF* and controls calculated by Fisher’s exact test (p < 0.001). Note that *Mef2c-AHF-Cre;Tbx1*^*flox/flox*^ embryos had additional phenotypes (three with double outlet right ventricle, DORV and one with tetralogy of Fallot, TOF, as indicated); (B) H&E histological sections of the heart of a control embryo at E14.5, with a typical ventricular septum is shown in the inset on the lower right part of the image. (C) *Tbx1 LOF* embryo with a PTA-VSD. (D) *Mef2c-AHF-Cre* lineage tracing by using a *GFP* reporter allele in a control embryo at E9.5 and a representative sagittal section is shown in E. (F) *Mef2c-AHF-Cre* lineage tracing in a *Tbx1 LOF* embryo and a representative sagittal section of the embryo is shown in G. DAPI fluorescent stain to visualize nuclei and identify the tissue is shown in blue. (H) *Mef2c-AHF-Cre* lineage quantification from the area shown in the inset in G for control and *Tbx1 LOF* embryos. (I) Detection of *Tbx1* and *β-catenin* by qRT-PCR in control and *Tbx1 LOF* embryos. Statistical significance of the difference in gene expression was estimated using two-tailed t-test, FC = fold change, p values < 0.05. Error bars = standard deviation (SD). Abbreviations: aorta (Ao), pulmonary trunk (PT), left atrium (LA), right atrium (RA), left ventricle (LV), right ventricle (RV), pharyngeal arch (PA), outflow tract (OFT), 1, 2 and 3 indicate the first, second and third pharyngeal arches (PA), respectively.

To determine a specific role of *Tbx1* in the *Mef2c-AHF-Cre* domain, we generated two different genotypes, *Mef2c-AHF-Cre/+;Tbx1*^*f/-*^ and *Mef2c-AHF-Cre/+;Tbx1*^*f/f*^. Embryos at E14.5 with both genotypes had a persistent truncus arteriosus (PTA) with complete penetrance (n = 50; Fig 2A). Most but not all had an accompanying ventricular septal defect (VSD; n = 30; Fig 2B and 2C), in contrast to *Tbx1*^*-/-*^ mutant embryos, which all has a PTA with a VSD. The PTA was observed as early as E12.5 (S1 Fig). Due to the similarity in phenotype (Fig 2A), the two genotypes were combined and further referred to as *Tbx1 LOF*. In *Tbx1 LOF* embryos at E9.5, the pharyngeal apparatus and individual arches within appeared grossly normal (Fig 2F–2G) compared to control littermates (Fig 2D and 2E). This is distinctly different as compared to the global *Tbx1*^*-/-*^ null mutant embryos or mesoderm specific *Tbx1* conditional loss of function embryos at this stage [32] that have a severely hypoplastic distal pharyngeal apparatus. This rules out extreme morphology defects, such as absence of neural crest cell populations, as being responsible for the presence of a PTA in *Tbx1 LOF* embryos. We also performed lineage tracing (Fig 2D–2G) and observed that the *Mef2c-AHF-Cre* lineage in the AHF was only slightly reduced in *Tbx1 LOF* embryos versus control littermates at E9.5 (Fig 2H). By *in situ* hybridization analysis, *Tbx1* expression was greatly reduced in *Tbx1 LOF* embryos (S1 Fig) and this was confirmed by qRT-PCR (Fig 2I). Cell proliferation and apoptosis in the *Tbx1 LOF* versus control embryos did not show any significant difference in the *Mef2c-AHF-Cre* lineage in the AHF region between groups at E9.5 (S2 and S3 Figs). This is different than what was previously found for *Tbx1*^*-/-*^ [33] or *Nkx2-5*^*Cre*^ [22] conditional mutant embryos, which have changes in proliferation and apoptosis. We suggest the improved appearance of the distal pharyngeal apparatus in *Tbx1 LOF* embryos is due to differences in the *Mef2c-AHF-Cre* recombination domain. In relation to *β-catenin*, we noted a slight decrease in *Tbx1* expression in *β-cat GOF* embryos (S4 Fig). This was consistent, although not as dramatic, as was found previously using a broader mesenchymal *Cre* driver (*Dermo*^*Cre*^) [28]. We found *β-catenin* mRNA is significantly increased in expression in the AHF of *Tbx1 LOF* embryos by qRT-PCR (Fig 2I).

### *β-catenin* and *Tbx1* conditional mutants have opposite roles in AHF muscle cell differentiation

**Fig 3:**
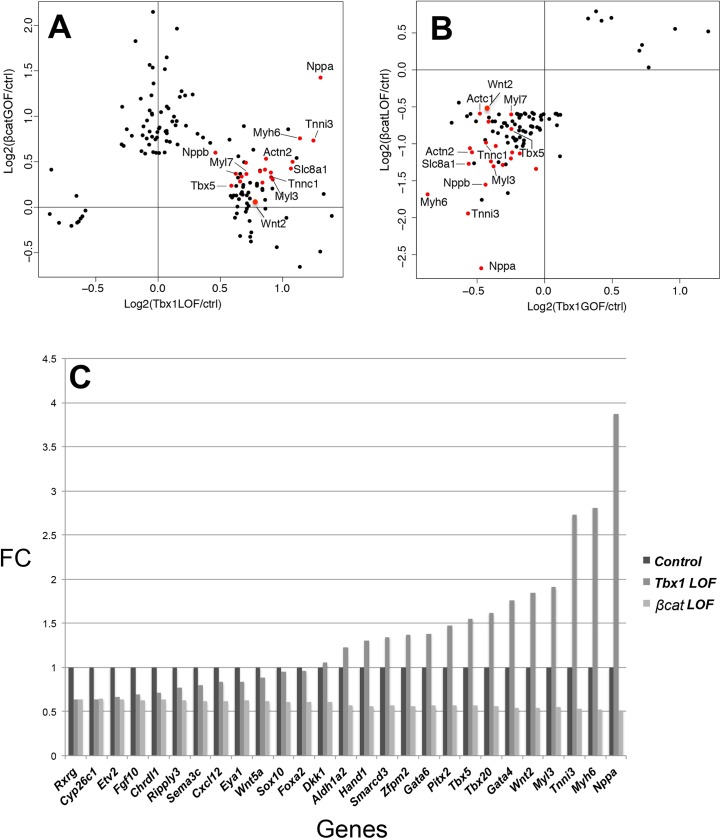
Opposing *β-catenin* and *Tbx1* conditional mutants have same effect on expression of pro-differentiation genes in the AHF. (A) Comparison of global gene expression changes in the micro-dissected AHF of *β-catenin GOF* and *Tbx1 LOF* embryos at E9.5. Plotted are differentially expressed genes (p < 0.05 and FC > 1.5) in at least one of the two comparisons, *Tbx1 LOF* vs controls (x-axis) or *β-catenin GOF* vs controls (y-axis). (B) Comparison of global gene expression changes in the micro-dissected AHF from *Tbx1 GOF* and *β-catenin LOF* embryos at E9.5. Plotted are differentially expressed genes (p < 0.05 and FC > 1.5) in at least one of the two comparisons, *Tbx1 GOF* vs controls (x-axis) or *β-catenin LOF* vs controls (y-axis). Red dots denote cardiac differentiation genes. (C) qRT-PCR analysis of *Tbx1 LOF* versus *β-catenin* LOF (FC = Fold Change) of selected genes demonstrating the opposite gene expression changes in these mutant embryos.

As for *β-catenin* gain of function in the AHF, we were interested in determining the function of loss and gain of *Tbx1* in the AHF. We previously generated a tissue specific constitutively expressing *Tbx1* gene [34]. Homozygous mice were crossed with *Mef2c-AHF-Cre* mice to overexpress *Tbx1* in the same domain as other alleles, and the embryos are referred to as *Tbx1 GOF*. Gene expression profiling of *Tbx1 LOF* and *GOF* embryos was performed of AHF tissue at E9.5, to test whether loss or gain of *Tbx1* would have opposing effects on muscle structural protein differentiation genes and to compare with findings of *β-catenin* loss and gain mutant embryos (Fig 3A–3C). The dot plots of global gene expression changes in the AHF between *β-cat GOF* versus *Tbx1 LOF* embryos showed increase in gene expression in the same direction (Fig 3A). The genes with the largest increase were the muscle structural protein genes. Similarly, *β-cat LOF* versus *Tbx1* GOF showed the same strong decrease of expression of muscle differentiation genes. The genes with the largest decrease were the muscle structural protein genes. These results provide functional genetic insight as to the previously implicated antagonistic relationship between *Tbx1* and *β-catenin* in the pharyngeal mesenchyme [28], that they perhaps are needed to balance cell differentiation. Some of the genes were tested by qRT-PCR for *Tbx1 LOF* and *β-cat LOF* embryos (Fig 3C). We found top genes decreased in expression in *Tbx1 LOF* embryos were not generally decreased in *β-cat LOF* embryos as top genes that were increased in expression. *Tbx1* was slightly increased in expression in *β-cat* LOF embryos (Fig 3C).

### Reduced *β-catenin* dosage significantly rescues heart defects in *Tbx1 LOF* embryos

**Fig 4:**
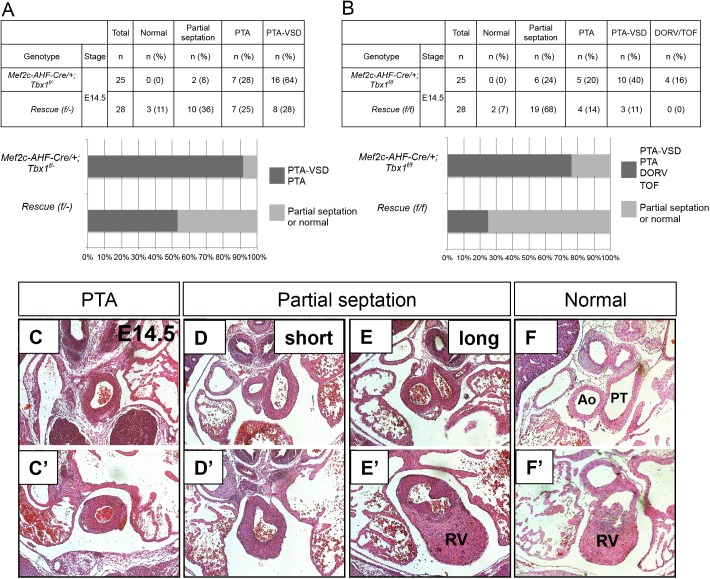
Phenotype analysis of significantly rescued *Tbx1 LOF* embryos. Phenotypes in embryos in which one *β-catenin* loss of function allele to either *Mef2c-AHF-Cre/+;Tbx1*^*f/-*^ embryos (A) or to *Mef2c-AHF-Cre/+;Tbx1*^*f/f*^ embryos (B) was done to lower the dosage of *β-catenin* within the AHF. Significant rescue (p < 0.001, Fisher’s exact test) was obtained in both sets of double mutant embryos. Middle panels show the percent within the groups with PTA (C and C’), DORVor TOF associated with a VSD or PTA without VSD (dark grey) and those ones with partial septation or a normal heart (light grey). Partial septation in *Tbx1 LOF* embryos means a PTA with presence of a short septum at some level of the OFT (D and D’) and complete septation between the ventricles (8/50) while partial septation in the rescue genotype embryos (34/56) means a range of noticeably less severe phenotypes including: a longer partial OFT septation (E and E’) and either complete septation between ventricles (15/56) or VSD (10/56) or normal OFT (F and F’) with a VSD (4/56) or normal heart [5/56] (F and F’). Abbreviations: aorta (Ao), pulmonary trunk (PT), right ventricle (RV), outflow tract (OFT), PTA (persistent truncus arteriosus).

**Fig 5:**
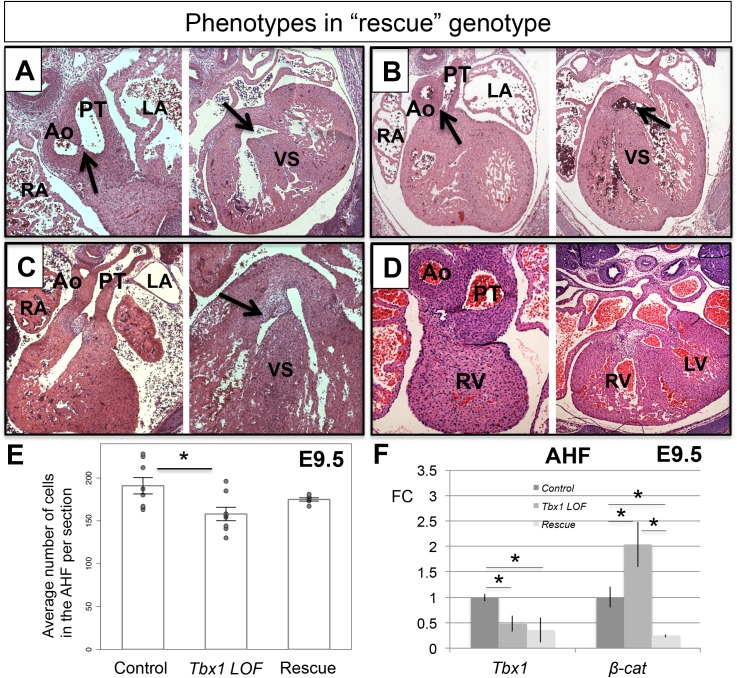
Histology analysis of representative embryos with the “rescue” genotype. Transverse H&E histological sections of hearts from E14.5 significantly rescued embryos (*Tbx1 LOF* with loss of one allele of *β-catenin* in the *Mef2c-AHF-Cre* domain). (A) An embryonic heart with a thin septum formed that separates the Ao and the PT (top black arrow), and a small VSD compared to the usual PTA-VSD in *Tbx1 LOF* embryos (Fig 2C). (B) Example showing a DORV (middle black arrow), with a separate Ao and PT; normal ventricular septation is present. (C) Example of a heart showing a partially rescued septation between the Ao and PT (middle black arrow) and normal septation between the two ventricles (right black arrow). (D) Rescued septation between the Ao and PT. (E) *Mef2c-AHF-Cre* lineage quantification from the same area shown in Fig 2G for control, *Tbx1 LOF* and rescued embryos. (F) Detection of *Tbx1* and *β-catenin* by qRT-PCR in control, *Tbx1 LOF* and rescued embryos. Statistical significance of the difference in gene expression was estimated using two-tailed t-test, FC = fold change, p values < 0.05. Error bars = standard deviation (SD). Abbreviations: RA = right atrium, RV = right ventricle, LA = left atrium, LV = left ventricle, Ao = Aorta, PT = pulmonary trunk, VS = ventricular septum, PTA = persistent truncus arteriosus, VSD = ventricular septal defect, DORV = double outlet right ventricle, OFT = outflow tract.

Based upon the opposing gene expression changes between *Tbx1* and *β-catenin* in the AHF, that also included *β-catenin* mRNA itself in *Tbx1 LOF* embryos (Fig 2I), we tested whether we could rescue heart defects in the *Tbx1* conditional loss of function mutant embryos by inactivating one allele of *β-catenin* in the *Mef2c-AHF-Cre* domain (*Mef2c-AHF-Cre/+;Tbx1*^*f/-*^ and *Mef2c-AHF-Cre/+;Tbx1*^*f/f*^). Details of the background and crosses are provided in the Methods section and details of the control genotypes are provided in S1 Table. Inactivation of one allele of *β-catenin* in the *Mef2c-AHF-Cre* domain did not result in any cardiovascular defects (S1 Table and [27]). Significant rescue (p < 0.001, Fisher’s exact test) was obtained in both sets of double mutant rescue genotype embryos (Fig 4). Upon combining all double mutant embryos together (n = 56), a total of 61% (n = 34/56) showed some rescue of the PTA phenotype (Fig 4). Specifically, complete distal OFT and partial proximal OFT septation and/or complete septation between the ventricles were present in these hearts (Fig 5A–5D). Ten percent showed complete rescue. Additional and more posterior sections can be found in S5 Fig Lineage tracing of the double mutant rescue genotyped embryos showed no significant difference in the number of cells in the AHF, of *Mef2c-AHF-Cre* lineage compared to the control or *Tbx1 LOF* embryos at E9.5 (Fig 5E). Finally, qRT-PCR was performed and *Tbx1* and *β-catenin* mRNA expression were reduced in the AHF of these embryos compared to the control (Fig 5F).

### Genes for cardiomyocyte differentiation are normalized in rescued embryos

**Fig 6:**
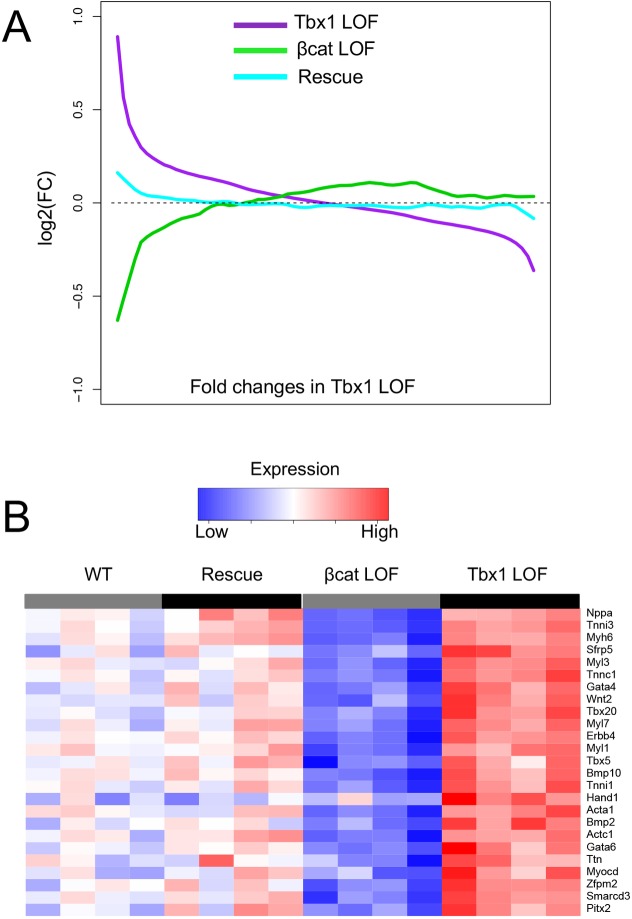
Gene expression of cardiac muscle differentiation genes are normalized in rescued embryos. (A) Comparison of global expression changes. Differentially expressed genes (p < 0.05; n = 3,636) in either *Tbx1 LOF* or *β-catenin LOF* were sorted by their FC between *Tbx1 LOF* and controls (x-axis). The sorted genes were grouped (50 genes per group) and then the average expression changes for each group in the *Tbx1 LOF*, *β-catenin LOF* or rescue embryos (vs controls) were plotted in the y-axis. (B) Heatmap showing the expression of selected key genes increased in expression in *Tbx1 LOF* embryos but decreased in expression in *β-catenin LOF* embryos.

Since we identified the greatest increase of expression in *Tbx1 LOF* and decrease in *β-cat LOF* embryos pertaining to muscle differentiation genes, we tested if there is global normalization of expression in the embryos of the double mutant, rescue genotype. For this test, gene expression profiling was performed on these embryos, in the same way for the individual mutant embryos and we found this to be the case. Expression of genes with greatest increase in *Tbx1 LOF* embryos (>1.3 fold), primarily the differentiation genes and greatest decrease in *β-cat LOF* embryos were largely normalized in rescued embryos (Fig 6A). However, we did not observe this same strong finding for genes increased in *β-cat LOF* embryos.

As mentioned, most of the genes with the strongest increase of expression in *Tbx1 LOF* and decrease in *β-cat LOF* embryos that were normalized (p<0.01) in rescued embryos, were genes that encode smooth or cardiac muscle genes (Figs 3C and 6B). This also included major transcription factors such as *Pitx2*, *Tbx5*, *Gata4* and *Gata6*, that are required for cardiac muscle differentiation [35–37]. The canonical Wnt gene, *Wnt2*, showed a similar pattern (Figs 3C and 6B). A full heatmap of the experiment is shown in S6 Fig Some additional genes of note, increased in expression in *Tbx1 LOF* embryos by gene profiling include *Myocd*, *Bmp2*, *Bmp10*, *Erbb4* and *Sfrp5*, which were oppositely affected in *β-cat LOF* embryos, and normalized in rescued embryos (Fig 6B and S6 Fig). This data supports the idea that pro-differentiation by canonical *Wnt/β-catenin* might be modulated by *Tbx1* in the *Mef2c-AHF-Cre* lineage.

Not all genes with increase in expression in *Tbx1 LOF* embryos and decrease in *β-cat LOF* showed normalization in rescued embryos (*Hand1*, *Zfpm2*, *Smarcd3* and *Tbx20*; Fig 6B and S7 Fig). Further, genes reduced in expression in both types of *LOF* mutants (S7 Fig) might not be relevant for the observed rescued phenotype since they were not normalized in rescue genotyped embryos. This suggests that other pathways are required for OFT development, and explains, in part, why complete rescue did not occur. Nonetheless it provides insights as to the nature of the relationship of the two genes, *Tbx1* and *β-catenin* as well as their independent functions.

## Discussion

### Tbx1 and β-catenin in the AHF

**Fig 7:**
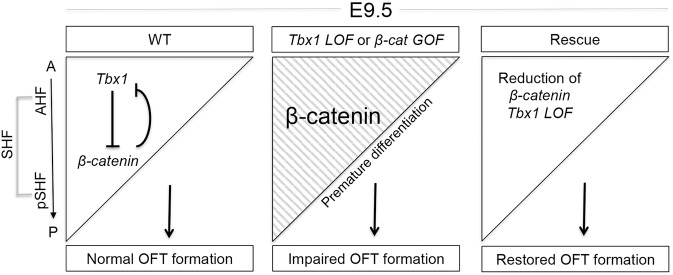
Model for Tbx1 and *β*-catenin function in the SHF. In the model, the triangle represents the *Mef2c-AHF-Cre* lineage and the *Tbx1* expression pattern, before migrating into the heart tube at E9.5. Left panel: *Tbx1* expression is strongest in the AHF and weakest in the posterior SHF (pSHF) while *Wnt/β-catenin* expression is opposite. Left panel depicts a possible double negative feedback loop between the two genes in the SHF, required for normal cardiac outflow tract (OFT) formation. Middle panel shows increased differentiation in the AHF when *Tbx1* is inactivated or *β-catenin* is constitutively active in the AHF. This results in premature differentiation within this tissue. Right panel depicts the rescue genotype in which both alleles of *Tbx1* and one allele of *β-catenin* was inactivated in the AHF. Significant rescue of heart defects was obtained. Abbreviations: A = anterior, P = posterior, OFT = outflow tract.

Loss of *β-catenin* using various pharyngeal mesoderm engineered *Cre* drivers, including *Mesp1-Cre* [38], *Nkx2-5-Cre* [39], *Isl1-Cre* [40, 41] and *Mef2c-AHF-Cre* [27] results in embryonic lethality due to the presence of cardiac outflow tract defects. The mechanisms mediating these abnormalities, in particularly within the pharyngeal mesoderm of the AHF, have not been well defined. This is especially important because there are many divergent and distinctive functions of β-catenin during cardiac development [42–45] [45] [38, 46, 47]. Our interest was to follow up on a previous study in which *Tbx1* expression was affected oppositely by loss or constitutively active *β-catenin* in the pharyngeal mesenchyme using a mesenchymal *Cre*, termed *Dermo*^*Cre*^ [28]. Based upon this finding, we investigated the two genes in the AHF tissue at stage E9.5, when the heart tube is elongating. We found that *β-catenin* promotes muscle differentiation in the AHF. We also found that *Tbx1* and *Wnt/β-catenin* act antagonistically to provide a balance of expression of pro-differentiation genes in the AHF that may be required for cardiac outflow tract development. This sheds new light onto the importance of the two genes in heart development as outlined in the model shown in Fig 7.

In the model in Fig 7, we illustrate the *Tbx1* expression domain in the SHF as a triangle, with the strongest expression anteriorly, in the AHF tissue and weakest in the posterior SHF. On the other hand, *Wnt/β-catenin* expression and function is strongest in the posterior SHF and weakest in the AHF, at E9.5 during mouse embryogenesis. In the panel on the left, we created a simple negative feedback loop, which is consistent with our findings in this study and previous findings using *Dermo*^*Cre*^ [28]. The center panel of the model illustrates the situation when *Tbx1* is inactivated or *β-catenin* is constitutively active in the AHF. Here, in these embryos differentiation occurs prematurely in the AHF, prior to reaching the heart tube (Fig 7, middle panel). This results in impaired cardiac outflow tract development.

In our study, we found that loss of *Tbx1* in the *Mef2c-AHF-Cre* domain, along with loss of one allele of *β-catenin* provided significant rescue of heart defects (Fig 7, right panel). This supports the importance of their interaction. However, rescue is not complete. An explanation for this is that *β-catenin* is only partially diminished when one allele is inactivated, such that complete normalization is not possible. Another explanation is that both *Tbx1* and *Wnt/β-catenin* act in many complex pathways in the AHF at this time point (E9.5), for which only the overlapping functions were normalized in rescued embryos [48–51]. Further, genes changing in expression at E9.5 may only be partially reflected in PTA defects observed at E12.5. We also note that the defects in the cardiac outflow tract between *Tbx1* loss and *β-catenin* gain in the *Mef2c-AHF-Cre* domain are different [27], supporting this idea. In particular, neonatal lethality occurs in *Tbx1 LOF* embryos due to the presence of a PTA, while gain of *β-catenin* results in mid-gestational lethality with a short, hypercellular outflow tract.

### Molecular mechanisms for *Tbx1* function

One of the main functions of *Tbx1* in the AHF is to maintain a progenitor state and restrict premature differentiation prior to reaching the elongating heart tube [22, 24]. Supporting this idea, constitutive overexpression of *Tbx1* in the *Mef2c-AHF-Cre* domain results in a decrease in expression of muscle differentiation genes [22]. Based upon our gene expression profiling data, we suggest that *Tbx1* may directly or indirectly, repress expression of key transcription factors that regulate this process i.e., it maintains the AHF cell fate. We suggest that there is a small decrease in AHF cell numbers but more importantly, a change in cell fate. Lack of observable morphology defects in the distal pharyngeal apparatus and lack of significant change in proliferation or apoptosis of the AHF progeny at E9.5 support this. Interestingly, in *Tbx1 LOF* mutant embryos, we found an increase in expression of genes required for cardiomyocyte specification, such as *Gata4*, *Tbx5* and *Smarcd3* (*Baf60c*) [52–55] [56–61] [35, 62–65]. Tbx5 and Gata4 proteins are co-expressed and physically interact to regulate expression of downstream muscle structural protein genes [52] [53]. The combination of *Tbx5*, *Gata4* and *Smarcd3* are sufficient to differentiate mouse embryonic mesoderm to beating cardiomyocytes [54]. Another intermediate protein is Serum Response Factor (SRF), which directly promotes expression of genes encoding muscle structural proteins that were found increased in *Tbx1* null mutant embryos [22]. Inactivation of *Tbx1* resulted in expansion of expression of SRF protein but not mRNA [22]. Similarly, we did not find *Srf* expression levels altered in *Tbx1 LOF* embryos. Of interest, the above transcription factors may interact with SRF protein to induce differentiation [66], supporting a continued role of SRF in *Tbx1* biology [22] [36, 67]. Of interest, the *Wnt2*, *Tbx5*, *Tbx20*, *Gata4* and *Gata6* genes are expressed and have function in the posterior SHF for formation of the inflow tract. It is not yet known if any are directly or indirectly regulated by *Tbx1*.

An additional role of *Tbx1* may be to restrict posterior SHF fate in the AHF so as to maintain the appropriate sub-populations within the SHF for proper heart development. We previously found expression of these posterior SHF genes were greatly expanded in the AHF tissue in *Tbx1* global null mutant embryos [24] and were increased in the same tissue in the conditional mutant embryos by qRT-PCR. It was previously found that *Wnt2* and *Gata6* act in the same genetic pathway in the posterior SHF during heart development and when inactivated cause atrial septal defects among other anomalies [68, 69]. We observed an increase in expression, but did not identify atrial septal defects. Since we did not observe a severe morphological defect in *Tbx1 LOF* embryos at E9.5, we suggest that some of these molecular changes will then affect later development.

### Relation of mouse developmental biology to 22q11.2 deletion syndrome

One of the challenges in human genetics is to identify risk factors of complex traits, such as congenital heart disease [70, 71]. The 22q11.2DS, although rare in the general population, offers a relatively homogenous cohort to investigate the basis of variable phenotypic heterogeneity among affected individuals. Rare deleterious DNA variants altered in expression in *Tbx1 LOF* embryos and acting antagonistically to canonical *Wnt/β-catenin*, might act as genetic modifiers of CHD in 22q11.2DS. Examination of whole exome sequence of 22q11.2DS subjects [72] is underway with a larger cohort, to identify such variants connected to *Tbx1* and *Wnt/β-catenin* gene networks or pathways needed to provide proper balance of critical cell fate choices. The work here provides a basis in the near future to translate efforts to studies of human subjects.

### Summary

In this study, we showed that inactivation of *Tbx1* in the *AHF* using *Mef2c-AHF-Cre* allele, results in a PTA that is also observed in the most seriously affected 22q11.2DS (velo-cardio-facial/DiGeorge syndrome) patients. The PTA defect in *Tbx1* conditional loss of function mutant embryos, was partially, but significantly rescued by decreasing one allele of the *β-catenin* gene in this domain, and this also resulted in a normalization of gene expression changes specifically for muscle differentiation but not necessarily for other classes of genes. Thus, we conclude that *Tbx1* in the *Mef2c-AHF-Cre* domain acts antagonistically with *Wnt/β-catenin* in the SHF to modulate differentiation prior to entering the heart tube.

## Methods

### Mouse mutant alleles

The following mouse mutant alleles used in this study have been previously described:

*Tbx1*^*+/-*^ [7], *Tbx1*^*f/+*^ (flox = f) [73], *Tbx1-GFP* [34], *β-catenin*^*f/+*^ and *β-catenin*^*E3/+*^ [29, 30], *Mef2c-AHF-Cre/+* [18], *ROSA26-GFP*^*f/+*^ (RCE:loxP)[74] and *Wnt/β-catenin* signaling reporter mice (Tg(TCF/Lef1-HIST1H2BB/EGFP)61Hadj/J; TCF/Lef:H2B-GFP [75]. To generate *Mef2c-AHF-Cre/+;Tbx1*^*f/-*^ mutant embryos (*Tbx1 LOF*), *Mef2c-AHF-Cre/+* transgenic male mice were crossed to *Tbx1*^*+/-*^ mice to obtain male *Mef2c-AHF-Cre/+;Tbx1*^*+/-*^ mice that were then crossed with *Tbx1*^*f/f*^ females. Alternatively, to generate *Mef2c-AHF-Cre/+;Tbx1*^*f/f*^ mutant embryos, *Mef2c-AHF-Cre/+* transgenic male mice were crossed to *Tbx1*^*f/f*^ mice to obtain male *Mef2c-AHF-Cre/+;Tbx1*^*f/+*^ mice, and these were then crossed with *Tbx1*^*f/f*^ females. Wild type and *Me2fc-AHF-Cre/+;Tbx1*^*f/+*^ littermates were used as controls for the experiments (First Tbx1 LOF and rescue crosses, S1 Table). *Tbx1* gain of function embryos (*Tbx1 GOF*) were generated by crossing male *Mef2c-AHF-Cre/+* mice with *Tbx1-GFP*^*f/f*^ females. To generate *Mef2c-AHF-Cre/+;β-catenin*^*f/f*^ mutant embryos (*β-cat LOF)*, male *Mef2c-AHF-Cre/+* transgenic mice were crossed to *β-catenin*^*f/f*^ females to obtain male *Mef2c-AHF-Cre/+;β-catenin*^*f/+*^ mice that were then crossed with *β-catenin*^*f/f*^ females. *β-catenin* gain of function (*β-cat GOF)* embryos i.e, *Mef2c-AHF-Cre/+;β-catenin*^*E3/+*^ or male *Mef2c-AHF-Cre/+* transgenic mice were crossed to *β-catenin*^*E3/E3*^ females. Double mutant embryos were generated by addition of one copy of the *β-catenin*^*flox*^ allele to *Tbx1 LOF* embryos resulting in what we denote as rescue genotyped embryos. In this case, the females used for the experimental crosses were of the *Tbx1*^*f/f*^*;β-catenin* ^*f/f*^ genotype, which have been maintained as an inbred line deriving from a mixed C57Bl/6; Swiss Webster background. The reporter *ROSA26-GFP* ^*f/+*^ allele was added to the *Tbx1*^*f/f*^ and *Tbx1*^*f/f*^*;β-catenin* ^*f/f*^ lines when visualizing *Mef2c-AHF-Cre* lineage. To evaluate *Wnt/β-catenin* signaling in wild type embryos, *TCF/Lef*:*H2B-GFP/+* reporter mice were used.

The *Mef2c-AHF-Cre/+;Tbx1*^*f/+*^ and the *Mef2c-AHF-Cre/+;Tbx1*^*+/-*^ mice are congenic in Swiss Webster. The *Tbx1*^*f/f*^*;β-catenin*^*f/f*^ mice are in an inbred line, as above. The *Mef2c-AHF-Cre/+;Tbx1*^*+/-*^ x *Tbx1*^*f/f*^*; β-catenin*^*f/f*^ crosses were performed 2 years before the *Mef2c-AHF-Cre/+;Tbx1*^*f/+*^ x *Tbx1*^*f/f*^*; β-catenin*^*f/f*^ crosses. The *Tbx1*^*f/f*^ and *ROSA26-GFP*^*f/+*^ lines are congenic in Swiss Webster. The *β-catenin*^*E3/+*^ and *β-catenin*^*f/f*^ mice were in a mixed C57Bl/6; Swiss Webster background. To exclude the possibility that a strain background might affect the possible rescue by *β-catenin LOF* allele, half of both *Tbx1 LOF* and the rescue genotyped embryos were generated by using *Tbx1*^*f/+*^*;β-catenin*^*f/+*^ females (second *Tbx1 LOF* and rescue crosses; S1 Table). Here, both *Tbx1 LOF* and the rescue genotyped embryos were littermates. The PCR strategies for mouse genotyping have been described in the original reports and are available upon request. All experiments including mice were carried out according to regulatory standards defined by the NIH and the Institute for Animal Studies, Albert Einstein College of Medicine (https://www.einstein.yu.edu/administration/animal-studies/), IACUC protocol # 2013–0405.

### Ethics statement

Institutional Animal Care and Use Committee (IACUC) approved this research. The IACUC number is 20160507.

### Whole mount *in situ* hybridization

Whole-mount RNA *in situ* hybridization with non-radioactive probes was performed as previously described [76, 77], using PCR-based probes, *Tbx1* [78], *Wnt2* forward primer: 5’ TGGCTCTGGCTCCCTCTGCT 3’ and reverse primer: 5’ CAGGGAGCCTGCCTCTCGGT 3’ and *Wnt4* forward primer: 5’ CCGCGAGCAATTGGCTGTACC 3’ and reverse primer: 5’ TGGAACCTGCAGCCACAGCG 3’. Following whole-mount protocol, the embryos were fixed overnight in 4% paraformaldehyde (PFA) and then dehydrated through a graded ethanol series, embedded in paraffin and sectioned at 10 μm. Minimum of 5 embryos from 3 independent litters were analyzed per embryonic stage.

### Proliferation and apoptosis on tissue sections

After fixation as described above, frozen sections were obtained at a thickness of 10 μm and then permeabilized in 0.5% Triton X-100 for 5 min. Blocking was performed with 5% serum (goat or donkey) in PBS/0.1% Triton X-100 (PBT) for 1 hour. Primary antibody was diluted in blocking solution (1:500) and incubated for 1 hour. Proliferation of cells was assessed by immunofluorescence using the primary antibody anti-phospho Histone H3 (Ser10), a mitosis marker (06–570 Millipore). Sections were washed in PBT and incubated with a secondary antibody for 1 hour. Secondary antibody was Alexa Fluor 568 goat a-rabbit IgG (A11011 Invitrogen) at 1:500. Slides were mounted in hard-set mounting medium with DAPI (Vector Labs H-1500). Images were captured using a Zeiss Axio Observer microscope. To perform statistical analysis of cell proliferation, we first counted the Mef2c-AHF-Cre, GFP positive cells in the pharyngeal apparatus located behind the heart in embryo sections and then calculated the average cell counts per tissue section for each embryo. Then we counted all proliferating cells in each section and calculated the ratio of proliferating cells within the Mef2c-AHF-Cre lineage. Then, we estimated the mean and standard error of the average cell counts for controls, *Tbx1 LOF* and rescued embryos and compared them using the t-test. Apoptosis was assessed on 10 μm thick frozen sections by using TMR Red *In situ* Cell Death kit (2156792 Roche) following the manufacturer’s instructions. Natural GFP from the reporter or an antibody for GFP (Abcam 6290) was used to distinguish the AHF cells in both assays described above. Representations of the complete AHF region from at least 4 embryos per genotype from at least 3 independent litters were used in each assay.

### *Wnt/β-catenin* signaling in mouse wild type embryos

*Wnt/β-catenin* signaling reporter mice, *TCF/Lef*:*H2B-GFP* [75] were used to observe *Wnt/β-catenin* signaling by direct fluorescence of green fluorescent protein (GFP) in wild type embryos at embryonic day E9.5 (19–21 somite pairs). Mouse embryos were fixed and cryosectioned at 10 μm. Slides were mounted in hard-set mounting medium with DAPI to visualize DNA (Vector Labs H-1500). Images were then captured using a Zeiss Axio Observer microscope. Nuclear *Wnt/β-catenin* signaling was counted as the GFP positive signal that co-localized with the DNA. A minimum of 5 embryos from 3 independent litters was analyzed.

### Mouse embryo heart histology and phenotypic analysis

Mouse embryos were isolated in phosphate-buffered saline (PBS) and fixed in 10% neutral buffered formalin (Sigma Corp.) overnight. Following fixation, the embryos were dehydrated through a graded ethanol series, embedded in paraffin and sectioned at 5 μm. All histological sections were stained with hematoxylin and eosin using standard protocols. Staining was performed in the Einstein Histopathology Core Facility (http://www.einstein.yu.edu/histopathology/page.aspx). For *Tbx1 LOF* mutants, a total of 70 hearts at E14.5 were obtained from more than 50 independent crosses and analyzed morphologically using light microscopy. For the rescue crosses, 56 hearts at E14.5 were obtained and the Fisher’s exact test was performed to compare the proportion of rescued phenotypes observed between rescued genotype hearts and the *Tbx1 LOF* mutants.

### Direct fluorescence: *Mef2c-AHF-Cre* lineage tracing

Images were generated from GFP expressing embryos by direct fluorescence immediately following dissection. For tissue sections, embryos were fixed for 2 hours with embryos stage ≤ E10.5 (30–32 somite pairs). Fixation was carried out in 4% PFA in PBS at 4°C. After fixation, tissue was washed in PBS and then cryoprotected in 30% sucrose in PBS overnight at 4°C. Embryos were embedded in OCT and cryosectioned at 10 μm. Images were then captured using a Zeiss Axio Observer microscope.

### Gene expression profiling on microarrays

Embryos at E9.5 (19–21 somites pairs) were used for global gene expression studies. To obtain enough RNA for microarray hybridization experiments, microdissected AHFs (defined here as: pharyngeal arches 2–6) from 27 of each of the following genotypes: *Tbx1 LOF* and its control (*Tbx1*^*f/+*^), *Tbx1 GOF* and its control (*Tbx1-GFP/+*), *β-cat LOF* and its control (*β-catenin*^*f/+*^), *β-cat GOF* and its control (*β-cat*^E3/+^), rescue and its control (*Tbx1*^*f/+*^;*β-catenin*^*f/+*^), were pooled in groups of three or six according to the genotype. For this experiment we used controls that did not have *Cre*. Between 4–6 microarrays were performed per genotype in 2–3 batches. The tissue was homogenized in Buffer RLT (QIAGEN). Total RNA was isolated with the RNeasy Micro Kit according to the manufacturer’s protocol. Quality and quantity of total RNA were determined using an Agilent 2100 Bioanalyzer (Agilent) and an ND-1000 Spectrophotometer (NanoDrop), respectively. Biotinylated single-stranded cDNA targets were amplified from 100 nanograms (ng) starting total RNA using the Ovation RNA Amplification System V2 and FLOvation cDNA Biotin Module V2 (NuGEN). A total of 3.75 mg of cDNA was hybridized to the GeneChip Test3 array (Affymetrix) to test the quality of the labeled target. Nucleic acid samples that passed quality control were then hybridized to the Affymetrix Mouse GeneST 1.0 chip. Hybridization, washing, staining and scanning were performed in the Genomics Core at Einstein (https://www.einstein.yu.edu/research/shared-facilities/cores/46/genomics/) according to the Affymetrix manual.

### Microarray data analysis

Data analysis was performed in the R statistical package. GeneChip data were pre-processed by the ‘oligo’ package [79], which implements Robust Multichip Average (RMA) algorithm with background correction, quantile normalization and gene level summarization [80]. Afterwards, for convenience of comparison, only probe-set assigned to genes were kept for subsequent analysis. Multiple probe-sets for the same genes were collapsed by “average” to obtain a single measurement per gene [81]. As some arrays were assayed in different batches, we performed UPGMA (unweighted pair group method with arithmetic mean) clustering of samples by transcriptomic profile similarities based on the Spearman correlation coefficients. This analysis indicated clear batch effects, especially for *β-cat LOF* and *Tbx1 LOF* data (data not shown). Hence, we applied ComBat, an efficient batch effect removal approach, to remove batch effects [82]. This analysis detected some individual arrays of poor quality that were then excluded. In the end, to keep a balance between controls and mutants, we analyzed 4 arrays per genotype. The ‘Limma’ package was used for determining differential expression [83]. To address the issue that adjustment of batch effect by any linear model based approach (including ComBat) can introduce a systematic correlation structure in the data, which may lead to exaggerated confidence in differential expression analysis [84], we accounted for this correlation in Limma by adding ‘blocking for batch’ in the model. In the end, genes with p-values < 0.05 were further explored. The microarray data has been deposited to the GEO database (accession number: GSE78125).

### Quantitative RT-PCR

Embryos at E9.5 (19–21 somites pairs) were used for quantitative gene expression studies of microdissected AHFs from each of the following genotypes: *Tbx1 LOF* and its control (*Tbx1*^*f/+*^), *Tbx1 GOF*, *β-cat LOF*, *β-cat GOF* and rescue were pooled in groups according to genotype. *Tbx1*^*f/+*^ was used as control. To obtain enough total RNA and minimize the variability of gene expression in individual embryos, each biological replicate of RNA contained microdissected AHFs from six embryos of the same genotype at E9.5 collected from at least 3 independent litters. Three biological replicates were performed per genotype. The tissue was immediately frozen, samples were homogenized and total RNA was isolated with the RNeasy Micro Kit (Qiagen). Quality and quantity of total RNA was determined using an Agilent 2100 Bioanalyzer (Agilent) and a ND-1000 Spectrophotometer (NanoDrop), respectively. Single-stranded cDNA targets were amplified from 100 nanograms (ng) starting total RNA using the Ovation RNA Amplification System V2 and FL- Ovation cDNA Biotin Module V2 (NuGEN). The mRNA levels were measured using TaqMan Gene Expression assays (Applied Biosystems) for each gene and were carried out in triplicate using *18S* (RNA, 18S ribosomal 1), *Actb* (Actin, beta) and *B2m* (Beta-2-microglobulin) genes as normalization controls. TaqMan probes and primer sets were obtained from the Applied Biosystems Gene Expression Assay database (http://allgenes.com). Samples were processed in standard 96-well plates (20 ul final volume per reaction and each reaction in triplicate containing 25 ng of cDNA) on an ABI 7900HT Q-PCR apparatus. The SDS 2.2 software platform (Applied Biosystems) was used for the computer interface with the ABI 7900HT PCR System to generate normalized data, compare samples, and calculate the relative quantity. Statistical significance of the difference in gene expression was estimated using ANOVA and the two-tailed t-test independently when type of comparison allowed it.

### Web resources

http://www.omim.org

http://genome.ucsc.edu/

http://www.R-project.org

## Supporting information

S1 Table Embryo genotypes used as controls for Tbx1 LOF and rescue experiments (Figs 2 and 4). The control embryo genotypes are listed in the first column. N is the number of embryos examined for heart and aortic arch anomalies. The % that is of a normal phenotype is indicated. Those with defects are identical to what was previously published as indicated.
(PDF)

S1 Fig *Tbx1* inactivation in *Tbx1 LOF* embryos. Whole mount *in situ* hybridization (WMISH) of *Tbx1* in a control embryo at embryonic day 9.5 (E9.5) and the corresponding sagittal section is shown in B. (C) WMISH of *Tbx1* in a AHF conditional mutant and the corresponding sagittal section is shown in D. 1 and 2 indicate the first and second pharyngeal arches, respectively. H&E histological sections of embryos at E12.5, control (E and F) and *Tbx1 LOF* (G and H). Abbreviations: heart (H), aorta (Ao), pulmonary trunk (PT) and persistent truncus arteriosus (PTA).
(PDF)

S2 Fig Proliferation of AHF in control, *Tbx1 LOF* and rescue embryos. (A) Immunofluorescence images of sagittal sections to visualize the AHF lineage (GFP, green) and cell proliferation (anti-phospho Histone H3 (Ser10); red); in control, *Tbx1 LOF* and rescue embryos are shown. DAPI fluorescent stain to visualize nuclei and identify the tissue is shown in blue. Statistical analysis was performed to determine whether cell proliferation was the same or different between groups of embryos by two-tailed t-test, p value <0.05. Error bars = standard deviation (SD). Abbreviations: outflow tract (OFT), right ventricle (RV).
(PDF)

S3 Fig Apoptosis of AHF in control, *Tbx1 LOF* and rescue embryos. (A) Immunofluorescence images of sagittal sections to visualize the AHF lineage (GFP, green) and apoptosis (TUNEL, red); in control, *Tbx1 LOF* and rescue embryos are shown. DAPI fluorescent stain to visualize nuclei and identify the tissue is shown in blue. Statistical analysis was performed to determine whether the number of dead cells was the same or different between groups of embryos by two-tailed t-test, p value <0.05. Error bars = standard deviation (SD. Abbreviations: outflow tract (OFT), right ventricle (RV).
(PDF)

S4 Fig Detection of *Tbx1* and *β-catenin* by qRT-PCR in control, *Tbx1 LOF* and *β-catenin GOF* embryos. Statistical significance of the difference in gene expression was estimated using two-tailed t-test, FC = fold change, p values < 0.05. Note that *β-catenin* mRNA is not expected to have a significant change in the AHF of *β-catenin GOF* embryos due to be a constitutive protein activation.
(PDF)

S5 Fig Posterior histology analysis of representative embryos with the “rescue” genotype. Additional transverse H&E histological sections of rescued hearts (*Tbx1 LOF* with loss of one allele of *β-catenin* in the *Mef2c-AHF-Cre* domain) at E14.5. (A—F) and (G–L) sections show two embryonic hearts with rescued septation between the two ventricles throughout the heart. Abbreviations: left atrium (LA), right atrium (RA), left ventricle (LV), right ventricle (RV).
(PDF)

S6 Fig Full heatmap of gene expression changes in Tbx1 LOF, *β-catenin* and rescue genotypes (Fig 6B). Heatmap showing the expression changes for all genes differentially expressed (p < 0.01) between *Tbx1 LOF*, *β-catenin LOF* or rescue embryos vs their respective controls.
(PDF)

S7 Fig Gene expression of cardiac morphogenesis genes. (A) Quantitative PCR was performed on micro-dissected *AHF of Tbx1 LOF*, *β-catenin LOF* and rescue embryos to detect the expression levels of selected genes known important for cardiac morphogenesis. *Tbx1*^*f/+*^ was used as control for expression plates due to number of genes tested and array design. Statistical significance of the difference in gene expression was estimated using ANOVA; p values < 0.05. Asterisks note those genes which expressions were, at least, significantly different between control and rescue embryos. FC = fold change. Error bars = standard deviation (SD).
(PDF)
