# Supplementary material for: Reduced dosage of β-catenin provides significant rescue of cardiac outflow tract anomalies in a Tbx1 conditional null mouse model of 22q11.2 deletion syndrome
Source: PLoS Genet. 2017 Mar 27;13(3):e1006687. doi: 10.1371/journal.pgen.1006687 (PMC5386301; doi:10.1371/journal.pgen.1006687)
Supplement: S1 Table — The control embryo genotypes are listed in the first column. N is the number of embryos examined for heart and aortic arch anomalies. The % that is of a normal phenotype is indicated. Those with defects are identical to what was previously published as indicated. (PDF) [file pgen.1006687.s001.pdf]

S1 Table (Related to Figs. 2 and 4)				
Embryo genotypes used as controls		Heart morphology		
	N	Normal (%)		
First Tbx1 LOF and rescue crosses				
Mef2c-AHF-Cre/+; Tbx1f/+; βcat f/+	8	100%		
Mef2c-AHF-Cre/+; Tbx1f/+	10	100%		
Second Tbx1 LOF and rescue crosses (to test for genetic background effects)				
Mef2c-AHF-Cre/+; Tbx1f/+; βcat f/+	3	100%		
Mef2c-AHF-Cre/+; Tbx1f/+	10	100%		
Tbx1 f/+; βcat f/+	4	100%		
Mef2c-AHF-Cre/+; βcat f/+	7	100%	*Same as Ai et al., 2007*	
Mef2c-AHF-Cre/+; βcat f/f	5	0%	*Same as Ai et al., 2007*	
βcat f/+	2	100%		
Mef2c-AHF-Cre/+; βcat E3/+	3	0%	*Same as Ai et al., 2007*	
βcat E3/+	4	100%	*Same as Ai et al., 2007*	
