# Supplementary figures and images for: Reduced dosage of β-catenin provides significant rescue of cardiac outflow tract anomalies in a Tbx1 conditional null mouse model of 22q11.2 deletion syndrome
Source: PLoS Genet. 2017 Mar 27;13(3):e1006687. doi: 10.1371/journal.pgen.1006687 (PMC5386301; doi:10.1371/journal.pgen.1006687)

S1 Fig. Related to Fig 2.

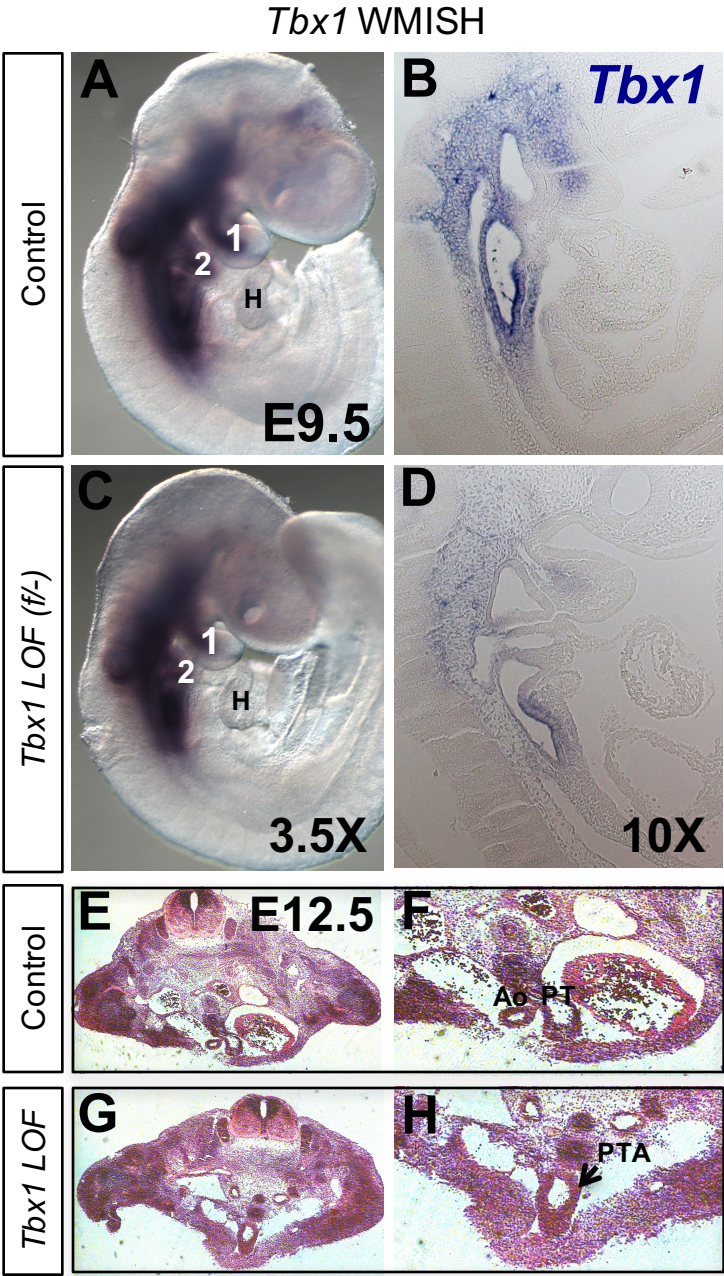

Supplement: S1 Fig — Whole mount in situ hybridization (WMISH) of Tbx1 in a control embryo at embryonic day 9.5 (E9.5) and the corresponding sagittal section is shown in B. (C) WMISH of Tbx1 in a AHF conditional mutant and the corresponding sagittal section is shown in D. 1 and 2 indicate the first and second pharyngeal arches, respectively. H&E histological sections of embryos at E12.5, control (E and F) and Tbx1 LOF (G and H). Abbreviations: heart (H), aorta (Ao), pulmonary trunk (PT) and persistent truncus arteriosus (PTA). (PDF) [file pgen.1006687.s002.pdf]

S2 Fig. Related to Figs. 2 and 5.

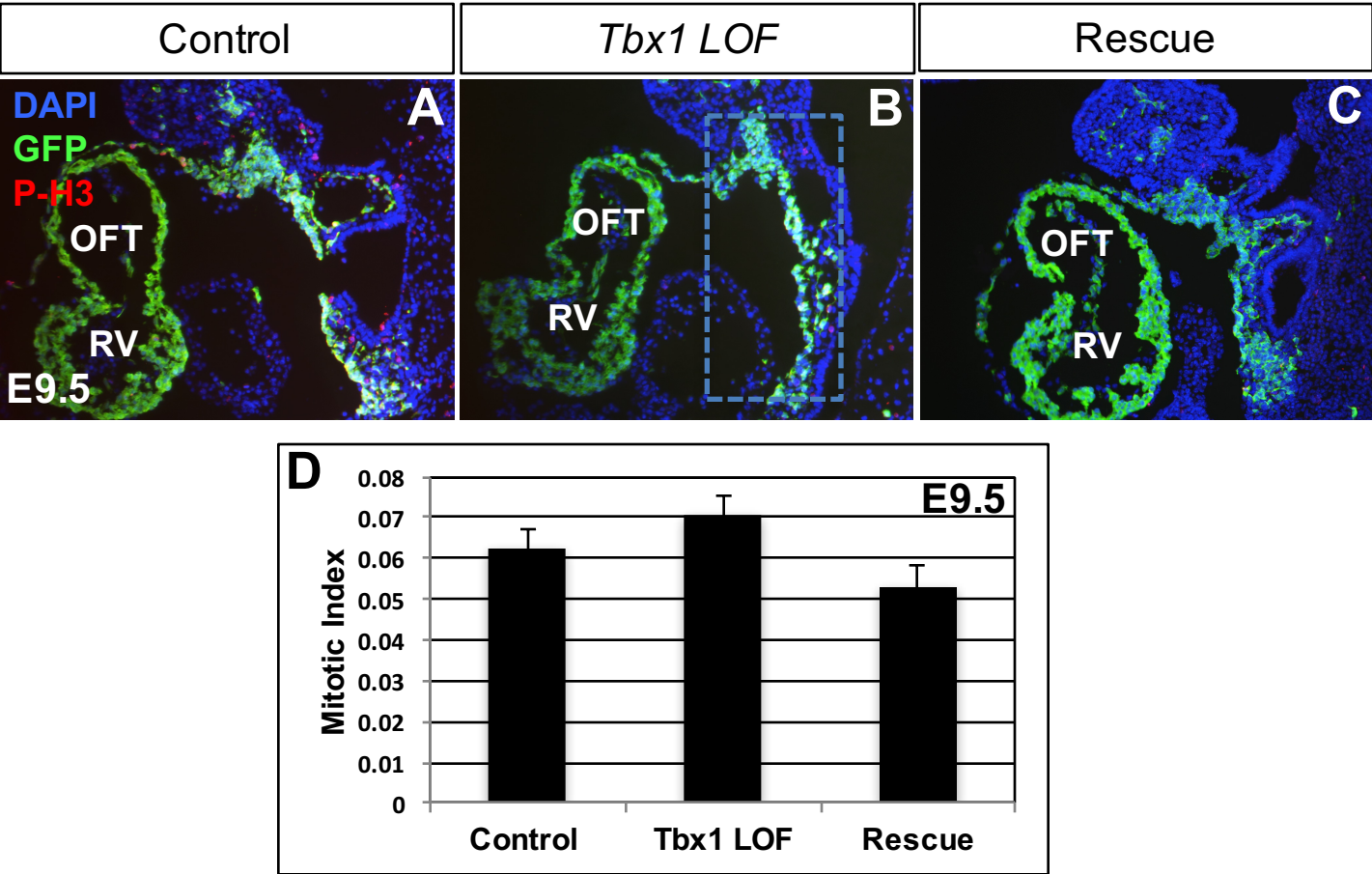

Supplement: S2 Fig — (A) Immunofluorescence images of sagittal sections to visualize the AHF lineage (GFP, green) and cell proliferation (anti-phospho Histone H3 (Ser10); red); in control, Tbx1 LOF and rescue embryos are shown. DAPI fluorescent stain to visualize nuclei and identify the tissue is shown in blue. Statistical analysis was performed to determine whether cell proliferation was the same or different between groups of embryos by two-tailed t-test, p value <0.05. Error bars = standard deviation (SD). Abbreviations: outflow tract (OFT), right ventricle (RV). (PDF) [file pgen.1006687.s003.pdf]

S3 Fig. Related to Figs 2 and 5.

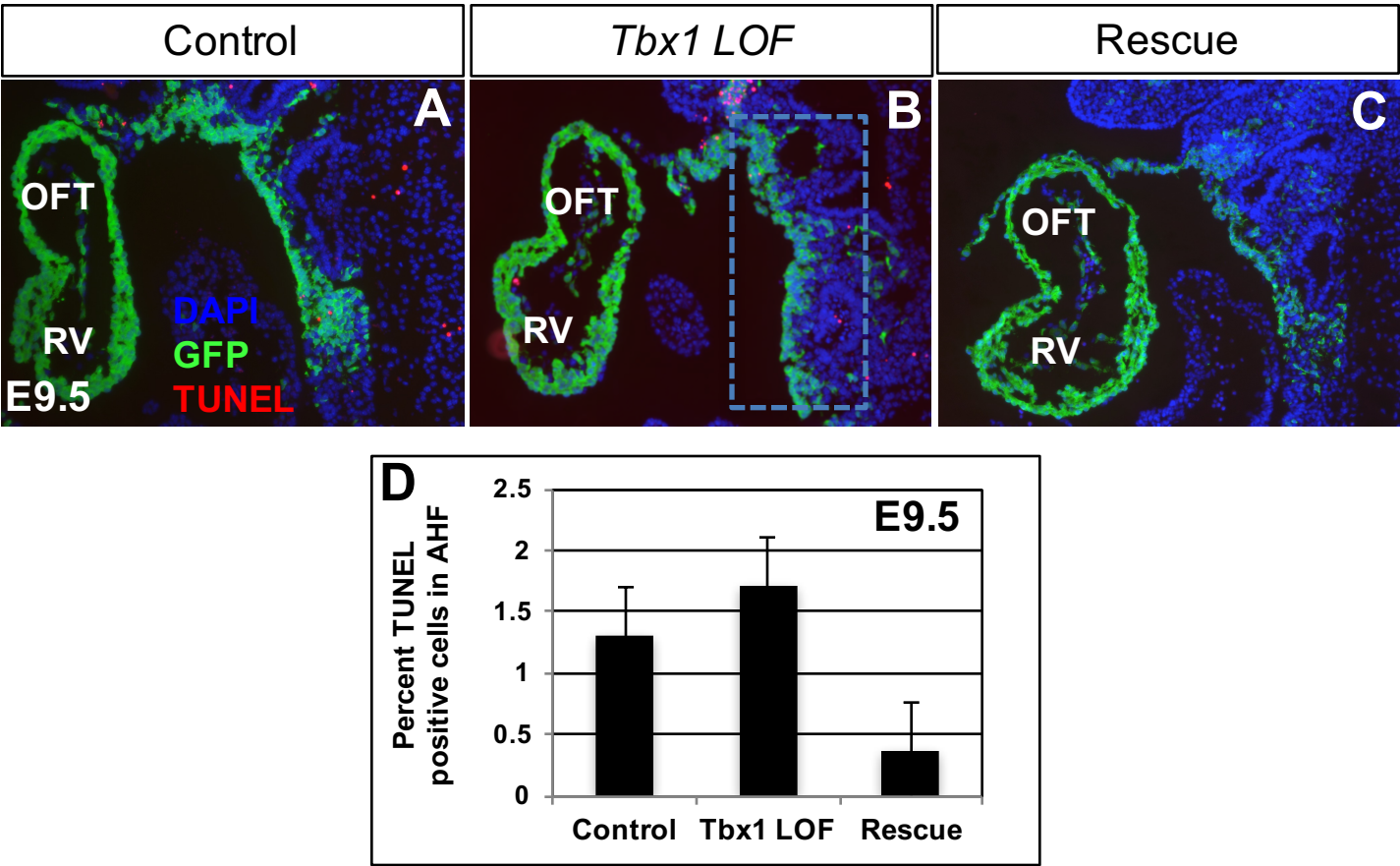

Supplement: S3 Fig — (A) Immunofluorescence images of sagittal sections to visualize the AHF lineage (GFP, green) and apoptosis (TUNEL, red); in control, Tbx1 LOF and rescue embryos are shown. DAPI fluorescent stain to visualize nuclei and identify the tissue is shown in blue. Statistical analysis was performed to determine whether the number of dead cells was the same or different between groups of embryos by two-tailed t-test, p value <0.05. Error bars = standard deviation (SD. Abbreviations: outflow tract (OFT), right ventricle (RV). (PDF) [file pgen.1006687.s004.pdf]

S4 Fig. Related to Fig 2 and Fig. 7.

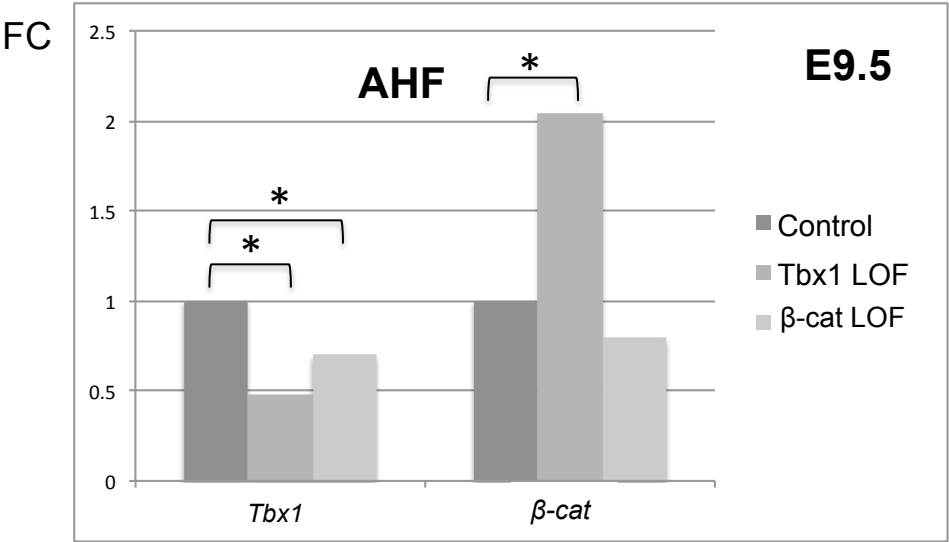

Supplement: S4 Fig — Statistical significance of the difference in gene expression was estimated using two-tailed t-test, FC = fold change, p values < 0.05. Note that β-catenin mRNA is not expected to have a significant change in the AHF of β-catenin GOF embryos due to be a constitutive protein activation. (PDF) [file pgen.1006687.s005.pdf]

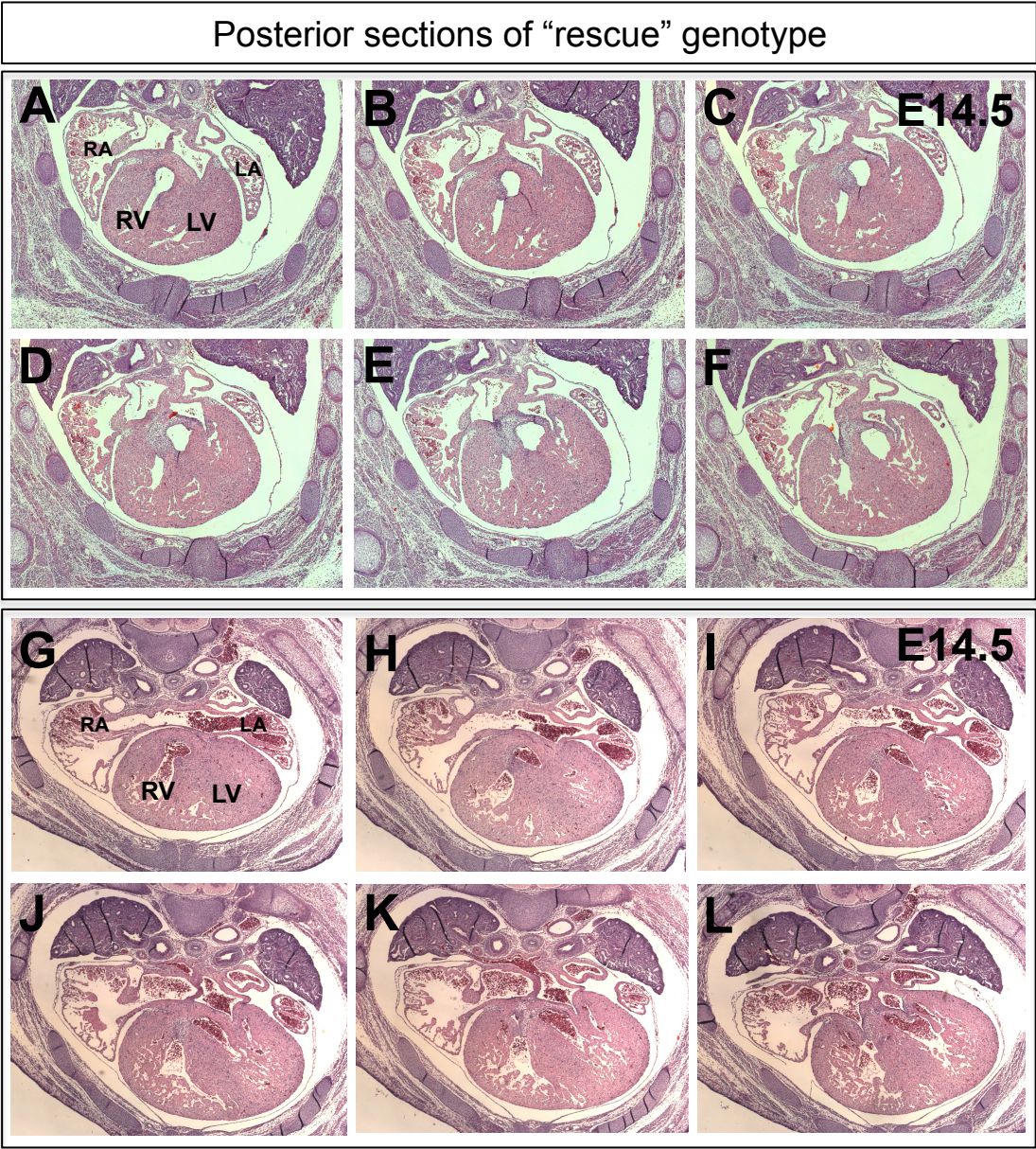

Supplement: S5 Fig — Additional transverse H&E histological sections of rescued hearts (Tbx1 LOF with loss of one allele of β-catenin in the Mef2c-AHF-Cre domain) at E14.5. (A—F) and (G–L) sections show two embryonic hearts with rescued septation between the two ventricles throughout the heart. Abbreviations: left atrium (LA), right atrium (RA), left ventricle (LV), right ventricle (RV). (PDF) [file pgen.1006687.s006.pdf]

S6 Fig. Related to Fig 6.

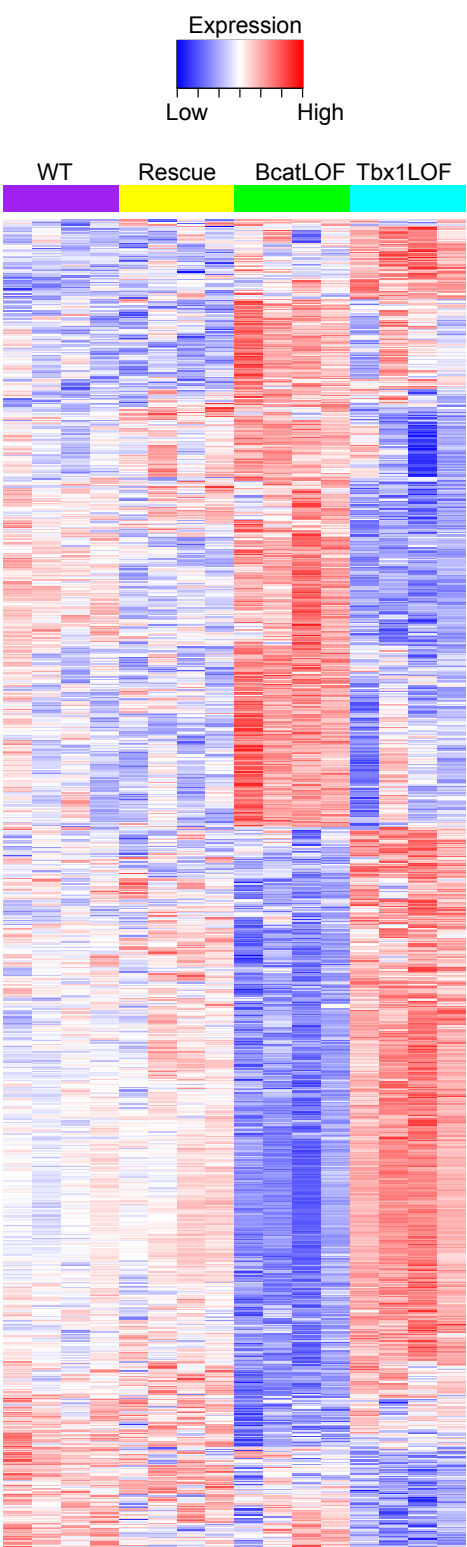

Supplement: S6 Fig — Heatmap showing the expression changes for all genes differentially expressed (p < 0.01) between Tbx1 LOF, β-catenin LOF or rescue embryos vs their respective controls. (PDF) [file pgen.1006687.s007.pdf]

S7 Fig. Related to Fig. 6.

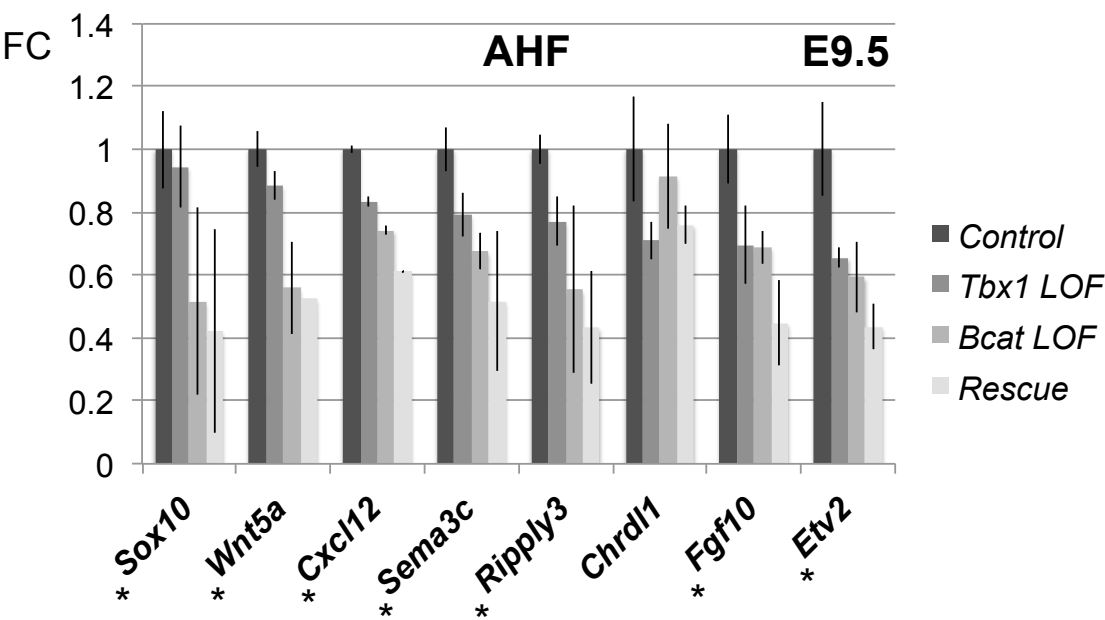

Supplement: S7 Fig — (A) Quantitative PCR was performed on micro-dissected AHF of Tbx1 LOF, β-catenin LOF and rescue embryos to detect the expression levels of selected genes known important for cardiac morphogenesis. Tbx1f/+ was used as control for expression plates due to number of genes tested and array design. Statistical significance of the difference in gene expression was estimated using ANOVA; p values < 0.05. Asterisks note those genes which expressions were, at least, significantly different between control and rescue embryos. FC = fold change. Error bars = standard deviation (SD). (PDF) [file pgen.1006687.s008.pdf]
